# Developing Story: No Link Observed between Prenatal PFOA/PFOS Exposure and Milestone Achievement

**Published:** 2008-10

**Authors:** Valerie J. Brown

Perfluorooctanoate (PFOA; also known as perfluorooctanoic acid) and perfluorooctanesulfonate (PFOS), chemicals used in waterproofing fabrics and greaseproofing fast-food containers, among other applications, have been detected at low concentrations in 98% of the U.S. population. These chemicals have half-lives of several years, and children tend to have higher serum concentrations than adults. Animal and human studies have hinted at a link between PFOA and PFOS and developmental effects, but human studies have been limited. A new human study suggests that maternal plasma levels of PFOA and PFOS may not be associated with delayed early development in babies **[*EHP* 116:1391–1395; Fei et al.]**.

The research team randomly selected 1,400 mother–baby pairs from the Danish National Birth Cohort comprising 100,000 women recruited during early pregnancy between 1996 and 2002. The team measured PFOA and PFOS levels in maternal blood samples taken in the first trimester of pregnancy. Each newborn’s Apgar score was obtained from Danish hospital records; this assessment of viability taken immediately after birth measures heart rate, respiratory effort, reflex irritability, muscle tone, and skin color.

When the children were 6 and 18 months old, their mothers provided data on gross and fine motor functioning, attention, cognitive function, language, and social-personal development via computer-assisted telephone interviews. The study was funded by the International Epidemiology Institute, which received money from the 3M Company, the original manufacturer of PFOA and PFOS, and the 3M Toxicology Laboratory performed the chemical analyses.

Earlier human and animal studies suggested that PFOA and PFOS might reduce fetal growth, delay learning, accelerate or delay sexual maturation, and produce other developmental effects. The current study found no significant association between maternal PFOA/PFOS levels and child achievement of early developmental milestones such as walking without support, taking off socks and shoes when asked, and turning a picture book right side up. However, a statistically nonsignificant association was observed between higher maternal PFOS levels and delay of a child’s ability to sit without support.

PFOA and PFOS were significantly higher among first-time mothers than in women who had given birth previously. This may confound their results, because the presence of older siblings may accelerate the developmental progress of younger ones. Given animal studies showing potential adverse effects of PFOA and PFOS, and the limited data for humans, the authors write that additional studies should be conducted using more sensitive measures of early childhood development.

